# Supplementary material for: Field-dependent specific heat of the canonical underdoped cuprate superconductor YBa2Cu4O8
Source: Sci Rep. 2020 Dec 18;10:22288. doi: 10.1038/s41598-020-79017-3 (PMC7749183; doi:10.1038/s41598-020-79017-3)
Supplement: Supplementary file 1 — Supplementary material 1 [file 41598_2020_79017_MOESM1_ESM.pdf]

Supplementary Information.

Field-dependent specific heat of the canonical underdoped cuprate superconductor $YBa_2Cu_4O_8$

Jeffery L. Tallon[1] and John W. Loram[2]

[1]Robinson Research Institute, and MacDiarmid Institute for Advanced Materials and Nanotechnology, Victoria University of Wellington, P.O. Box 33436, Lower Hutt 5046, New Zealand. jeff.tallon@vuw.ac.nz

[2]Cavendish Laboratory, Cambridge University, Cambridge CB3 0HE, United Kingdom.

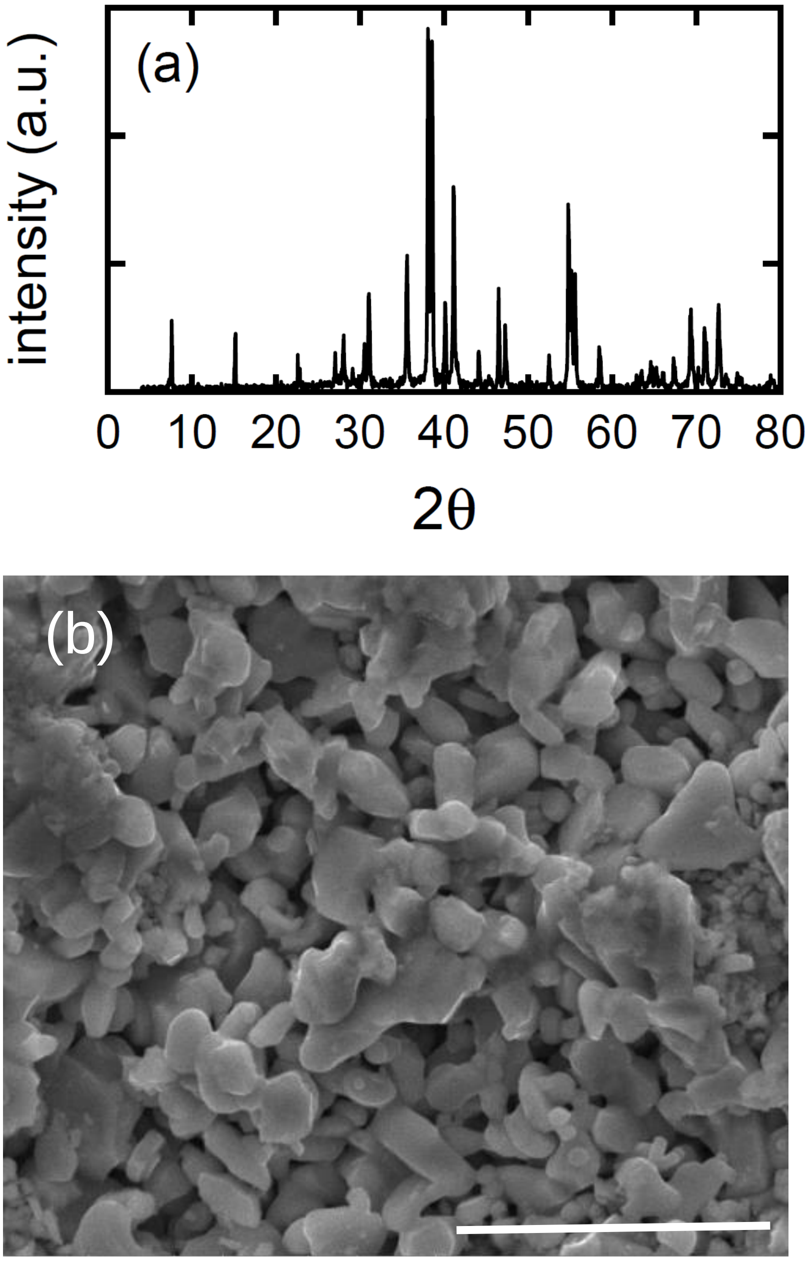

Figure S1 (a) typical x-ray diffraction pattern for the $YBa_2Cu_4O_8$ polycrystalline samples showing essentially single-phase composition (Co-Kα radiation). (b) SEM micrograph of the surface of a sample pellet. The white bar indicates 10 μm scale.

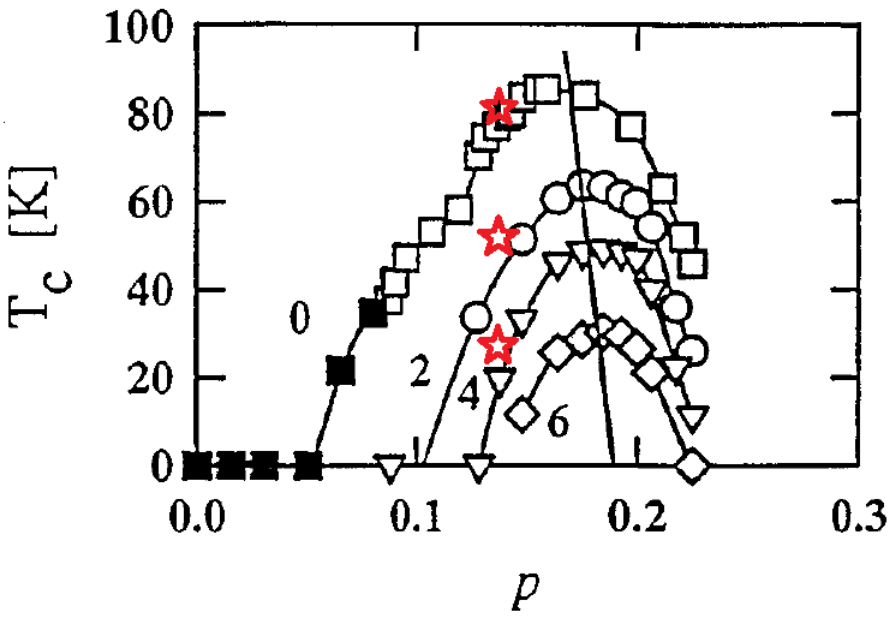

Figure S2. Black symbols: T_c versus hole concentration for $Y_{0.8}Ca_{0.2}Ba_2Cu_3O_{7-\delta}$ for 0, 2, 4 and 6% planar Zn concentration[1]. Red stars: T_c values for $YBa_2Cu_4O_8$ for 0, 2 and 4 % planar Zn concentration (this work).

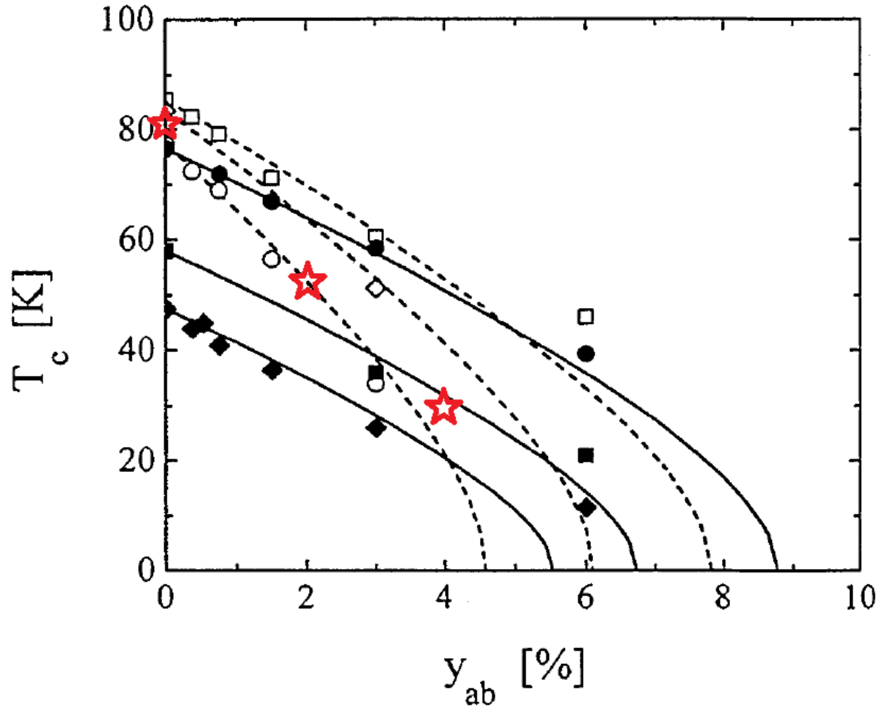

Figure S3. Black symbols: T_c versus planar Zn concentration[1] for $Y_{0.8}Ca_{0.2}Ba_2Cu_3O_{7-\delta}$. Solid curves and symbols: overdoped. Dashed curves and open symbols: underdoped. Red stars: T_c versus planar Zn concentration for $YBa_2Cu_4O_8$ (this work).

Comparison of ^{89}Y Knight shift with electronic entropy

The ^{89}Y Knight shift, $^{89}K_s$, for $YBa_2Cu_3O_{6+x}$ is reported by Alloul *et al.*[2] To convert to entropy units we must first convert to the spin susceptibility, χ_s. This was done by Alloul by comparing the temperature dependence of $^{89}K_s$ with that of the bulk magnetic susceptibility, χ_m. We use this relationship. The comparison of the *T*-dependent components is robust, however, each of $^{89}K_s$ and χ_m has an additive constant that must be identified if a comparison of absolute values is to be undertaken. For $^{89}K_s$ this additive constant is the chemical shift, $^{89}\sigma$, which is evaluated by Alloul as ranging from -200 ppm for x = 0.41 to -370 ppm for x = 1. In contrast Takigawa *et al.*[3] evaluate $^{89}\sigma$ as -152 ± 10 ppm independent of *x*. Our analysis below is consistent with this value, independent of *x*. This is the value that we also used[4] for $^{89}\sigma$ in $YBa_2Cu_4O_8$. For χ_m, the additive constant, χ_0, comprises a diamagnetic term and a van Vleck term ($\chi_0 = \chi_{dia} + \chi_{vv}$) estimated by Alloul as $\chi_{dia} = -2.65 \times 10^{-7}$ emu/g and $\chi_{vv} = 1.95 \times 10^{-7}$ emu/g, i.e. $\chi_0 = -0.7 \times 10^{-7}$ emu/g.

In view of the uncertainty of these *T*-independent parts we simply convert $^{89}K_s$ to χ_m using Allouls' Fig. 4 and multiply by a_W, as plotted in Fig. S4, for comparison with *S*/*T* for $YBa_2Cu_3O_{6+x}$. The $a_W\chi_m$ values for each *x* value were then displaced vertically (by an additive constant) to coincide with the entropy data. The first thing to note is that the *T*-variation of the susceptibility and entropy data for each specific value of *x* are in excellent agreement. Now if we take the value of this additive constant and work back to the chemical shift σ_0 we obtain values that vary quite narrowly between –130 and –150 ppm, very consistent with Takigawa[3]. This baseline uncertainty of ± 10 ppm corresponds to ± 0.04 mJ/g.at.K^2 in Fig. S4 and is rather small.

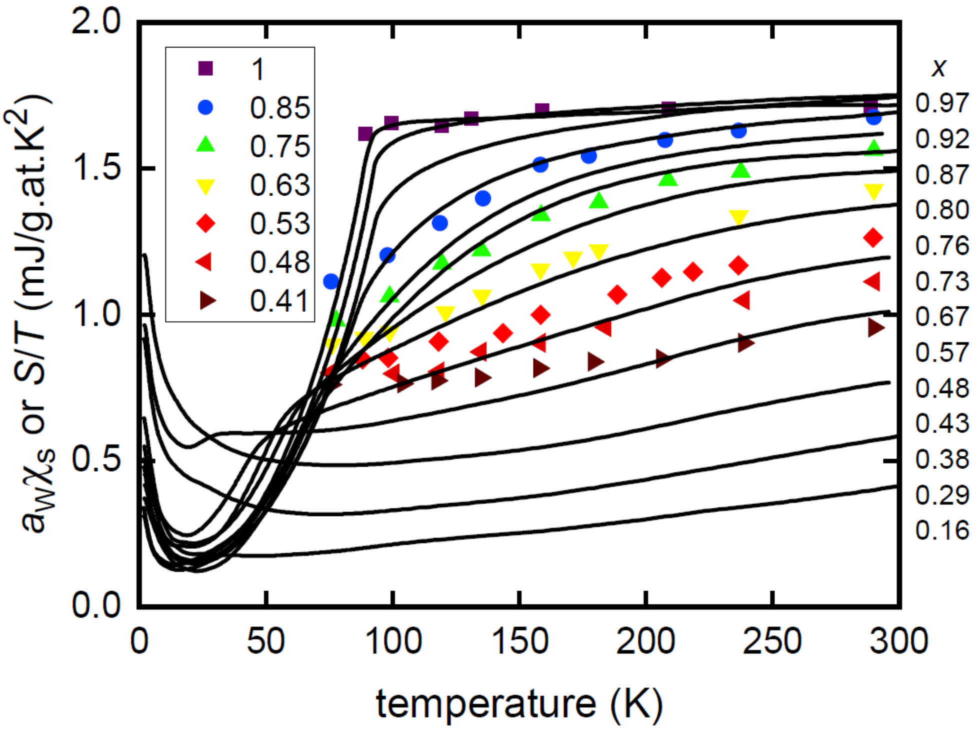

Figure S4. Data points: spin susceptibility for $YBa_2Cu_3O_{6+x}$ from the ^{89}Y Knight shift (reported by Alloul[2]) multiplied by the Wilson ratio in order to express in entropy units. *x* values are annotated. Solid curves: electronic entropy divided by *T* as reported by Loram *et al.*[5,6]

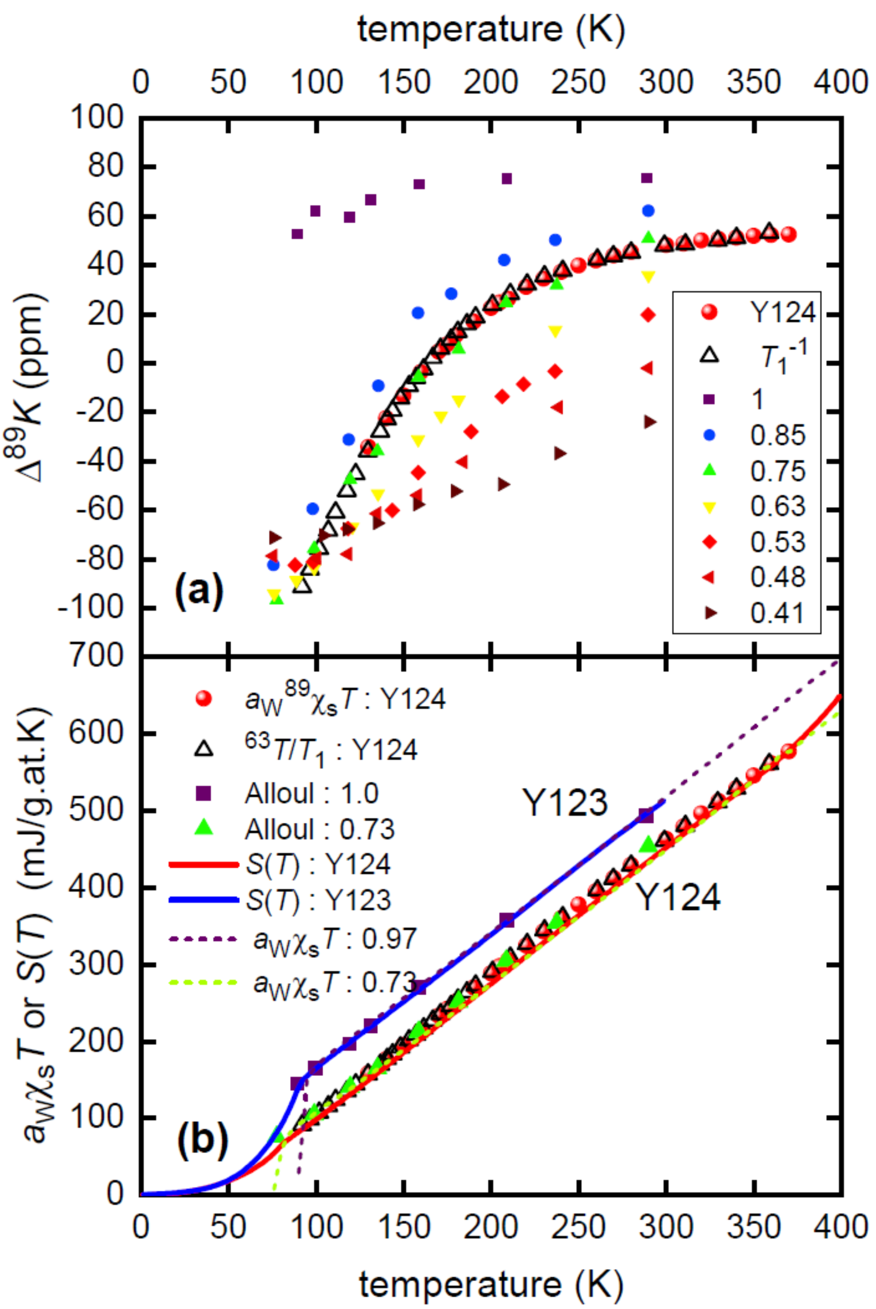

Figure S5. A reproduction of Fig. 7 but with bulk susceptibility data, $a_W\chi_sT$, (green dashed curve) overlayed on top of the entropy data, $S(T)$, (red solid curve). In Fig. 7 the susceptibility data was hidden by the entropy data. Here it is evident that the two agree closely over the entire temperature range.

References:

1. Tallon, J. L., Bernhard, C., Williams, G. V. M. & Loram, J. W. Zn-induced T_c Reduction in High-T_c Superconductors: Scattering in the Presence of a Pseudogap. *Phys. Rev. Lett.* **79**, 5294-5297 (1997).
2. Alloul, H., Ohno, T. & Mendels, P. ^{89}Y NMR evidence for a Fermi-liquid behavior in $YBa_2Cu_3O_{6+x}$. *Phys. Rev. Lett.* **63**, 1700-1703 (1989).
3. Takigawa, M., Hults, W. L. & Smith, J. L. *Phys. Rev. Lett.* **71**, 2650-2653 (1993).

4. Williams, G. V. M., Tallon, J. L., Meinhold, R. & Jánossy, A. ^{89}Y NMR study of the effect of Zn substitution on the spin dynamics of $YBa_2Cu_4O_8$. *Phys. Rev. B* **51** 16503-16506 (1995).
5. Loram, J. W., Mirza, K. A., Wade, J. M., Cooper, J. R. & Liang, W. Y. The Electronic Specific Heat of Cuprate Superconductors. *Physica C* **235-240**, 134-137 (1994).
6. Loram, J. W., Mirza, K. A. & Cooper, J. R. Properties of the superconducting condensate and normal-state pseudogap in high-T_c superconductors derived from the electronic specific heat. *IRC in Superconductivity Research Review Cambridge University* (1998).
